# myChEMBL: a virtual machine implementation of open data and cheminformatics tools

**DOI:** 10.1093/bioinformatics/btt666

**Published:** 2013-11-20

**Authors:** Rodrigo Ochoa, Mark Davies, George Papadatos, Francis Atkinson, John P. Overington

**Affiliations:** European Molecular Biology Laboratory, European Bioinformatics Institute (EMBL-EBI), Wellcome Trust Genome Campus, Hinxton CB10 1SD, UK

## Abstract

**Summary:** myChEMBL is a completely open platform, which combines public domain bioactivity data with open source database and cheminformatics technologies. myChEMBL consists of a Linux (Ubuntu) Virtual Machine featuring a PostgreSQL schema with the latest version of the ChEMBL database, as well as the latest RDKit cheminformatics libraries. In addition, a self-contained web interface is available, which can be modified and improved according to user specifications.

**Availability and implementation:** The VM is available at: ftp://ftp.ebi.ac.uk/pub/databases/chembl/VM/myChEMBL/current. The web interface and web services code is available at: https://github.com/rochoa85/myChEMBL.

**Contact:** jpo@ebi.ac.uk

## 1 MOTIVATION

The past decade has seen a dramatic increase in the number of informatics tools enabling the efficient analysis of the large chemical, biological and clinical datasets currently being produced by both academic institutions and pharmaceutical companies in the context of drug discovery (Wegner *et al.*, 2012). This was partly catalyzed by the development of freely accessible repositories containing expertly curated and annotated medicinal chemistry and chemical biology data. For example, the ChEMBL database is a public domain chemogenomics resource, which provides experimental data about the interactions between small molecules, including approved drugs and clinical candidates, and specific biological targets reported in the primary medicinal chemistry literature (Gaulton *et al.*, 2012). To facilitate the manipulation of such structure–activity relationship data, the integration of chemical information tools is required. These tools could then be a part of larger data mining protocols. Currently, a limited number of open source cheminformatics packages are available, while, more importantly, few of them incorporate chemical searching functionality using relational database architectures (i.e. a chemistry cartridge). RDKit (RDKit, 2013) offers such functionality within a PostgreSQL environment. Based on the easy integration of these open tools and data, a Virtual Machine was configured, and is ready to be used by both expert users who can write and execute custom SQL queries, and new users who can take advantage of a self-contained and user-friendly interface.

## 2 DATABASE CONFIGURATION

### 2.1 ChEMBL data configuration

The ChEMBL database was originally implemented as an Oracle schema. In addition, a MySQL snapshot is generated and distributed with each new version of the database. However, owing to RDKit’s requirements, a PostgreSQL version of ChEMBL was also implemented using the tools provided by the Ora2Pg project (http://ora2pg.darold.net/). The migration process was exhaustively tested to avoid loss of information or undesired changes over the data.

### 2.2 RDKit chemical cartridge functions

The RDKit chemical cartridge is written in C++ and, as a result, its chemical search performance is scalable given the large amount of structures currently stored in the ChEMBL database (∼1.2 million). With regard to the core functionality, the user can execute chemical searches based on substructure and similarity. In addition, the cartridge is capable of calculating physicochemical properties for a given molecule, such as logP, molecular weight and number of rings.

### 2.3 Database and virtual machine set up

**Fig. 1.**
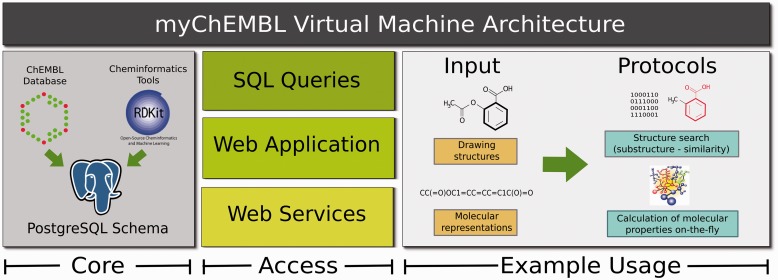
General architecture of the myChEMBL Virtual Machine. The VM core consists of all the bioactivity and chemical data stored in the ChEMBL database together with the cheminformatics tools provided by the RDKit chemical cartridge. Both are integrated through a PostgreSQL schema (version 9.1). All the protocols can be accessed using SQL queries, through a self-contained web interface or using RESTful web services. The basic usage needs as input a valid molecule using in-line formats or through sketchers. The output formats will vary depending on the access route chosen by the user

Initially, three tables were added to the existing ChEMBL schema. The first one contains the entire set of parent molecules converted to the RDKit format and appropriately indexed, using as input the original set of MDL Molfiles (Dalby *et al.*, 1992) stored in the database. The second table contains several types of molecular fingerprints per compound. The third table contains RDKit pre-generated depictions of the parent molecules stored in the first additional table. Having configured the database, a Virtual Machine was set up using the Ubuntu 12.10 64-bit operating system. It contains the latest ChEMBL PostgreSQL 9.1 schema with the aforementioned additions. The RDKit cartridge, along with all its requirements, was compiled and installed using the latest source code (version 2013-04). Moreover, documentation has been provided to aid and enhance the user experience. The VM can be easily imported using the freely available VirtualBox application (https://www.virtualbox.org). The general architecture is explained in Figure 1.

## 3 myChEMBL ACCESS

After downloading and installing the Virtual Machine, myChEMBL can be accessed in a number of ways. The user has the option to directly execute SQL queries based on the ChEMBL schema and the RDKit cartridge extensions. Alternatively, the web interface provided with the VM can be used locally. The final option is to install the VM as a remote web server, accessible to all users within an organization or project team, thus taking advantage of both the web applications, as well the web services provided.

### 3.1 Web application

A web-based application, developed using PHP, gives users without any prior knowledge of SQL, the ability to run searches against the myChEMBL system in a web browser. With regard to the user input, the query structures can be represented in three different formats. First, SMILES, which is the most common in-line representation of small molecules (Weininger, 1988). Second, MDL Molfiles or SMARTS queries for more advanced queries of chemical patterns; notably, the latter functionality is not currently offered by the main ChEMBL web interface (https://www.ebi.ac.uk/chembl/). Finally, the user has the option to draw the structure query using the open source sketcher JSME (Bienfait and Ertl, 2013), designed purely in JavaScript code.

### 3.2 Web services

The web application also provides a set of RESTful Web Services, which enable a user to programmatically access the core functionality offered by the web application. For example, the user can specify a similarity query through a URI format, with the option to select the desired molecular format, choose between a set of molecular fingerprints and additionally select (in the case of similarity searches) which similarity coefficient wants to be used for calculating the final scores. The services can be embedded in any programming language and workflow tools with available libraries for manipulating URL and JSON responses. A python client is provided with documented use cases for all the current functionalities.

## 4 FURTHER WORK

The original aim of the myChEMBL project was to build an open system, which would remove the technical burden of setting up a cheminformatics infrastructure, making it easier for users to interrogate in full the wealth of chemical and biological data stored within the ChEMBL database. The first release of myChEMBL is primarily focused on exploring the chemical space available within the ChEMBL database. Future functionality may include combining structure searches with the stored bioactivity data, to detect activity cliffs or matched molecular pairs in certain assays or biological targets. One of the more exciting opportunities would be to fully expose the ChEMBL data model, allowing users to load and curate their own datasets. The availability of a completely free self-contained version of ChEMBL (and a framework for loading analogous data) will hopefully catalyze further innovation and development in emerging economies, open innovation/community projects, e.g. in areas such as malaria and tuberculosis research. Finally, owing to the open philosophy of this project, we encourage the community to provide feedback, new ideas or code snippets, to enhance and improve the current functionality.

## References

[btt666-B1] Bienfait B, Ertl P (2013). JSME: a free molecule editor in JavaScript. J. Cheminform..

[btt666-B2] Dalby A (1992). Description of several chemical structure file formats used by computer programs developed at Molecular Design Limited. J. Chem. Inform. Comput. Sci..

[btt666-B3] Gaulton A (2012). ChEMBL: a large-scale bioactivity database for drug discovery. Nucleic Acids Res..

[btt666-B4] http://www.rdkit.org.

[btt666-B5] Wegner JK (2012). Cheminformatics. Commun. ACM.

[btt666-B6] Weininger D (1988). SMILES, a chemical language and information system. J. Chem. Inform. Comput. Sci..

